# Genetic Risk for Alcoholic Chronic Pancreatitis

**DOI:** 10.3390/ijerph8072747

**Published:** 2011-06-30

**Authors:** Marianges Zadrozny Gouvêa da Costa, Dulce Reis Guarita, Suzane Kioko Ono-Nita, Denise Cerqueira Paranaguá-Vezozzo, Guilherme Eduardo Gonçalves Felga, Martha Regina Arcon Pedroso, Marcelo Moreira Tavares de Souza, Paulo Dominguez Nasser, Camila da Silva Ferreira, Flair José Carrilho

**Affiliations:** Department of Gastroenterology, School of Medicine, University of São Paulo, São Paulo 05403-000, Brazil; E-Mails: d.guarita@uol.com.br (D.R.G.); skonita@gmail.com (S.K.O.-N.); denise.paranagua@gmail.com (D.C.P.-V.); uilherme.felga@gmail.com (G.E.G.F.); marthapedroso@yahoo.com.br (M.R.A.P.); marcelo_lim07@yahoo.com.br (M.M.T.S.); pdnasser@gmail.com (P.D.N.); camila.fr@usp.br (C.S.F.); fjcarrilho@hcnet.usp.br (F.J.C.)

**Keywords:** pancreatic diseases, alcoholism, genetics, cationic trypsinogen, cystic fibrosis transmembrane conductance regulator, serine protease inhibitor kazal type 1, chymotrypsin C

## Abstract

In recent years many studies have examined the genetic predisposition to pancreatic diseases. Pancreatic disease of an alcoholic etiology was determined to be a multi-factorial disease, where environmental factors interact with the genetic profile of the individual. In this review we discuss the main results from studies examining the frequency of genetic mutations in alcoholic chronic pancreatitis.

## 1. Epidemiology and Pathogenesis of Alcoholic Chronic Pancreatitis

Chronic pancreatitis is currently defined as permanent damage to the pancreatic gland, which leads to organ failure. Clinically it is associated with chronic abdominal pain, weight loss, steatorrhea, and diabetes. Affected individuals experience considerable economic losses due to reduced productivity, with consequent reduction in wages; frequent demand for health care; hospitalization; use of large quantities of drugs, including narcotics; and the possible need for surgical and therapeutic endoscopic procedures [1]. Knowledge of the epidemiology and pathogenesis are important for the prevention and progression of this disease [2].

The most commonly encountered cause of chronic pancreatitis is excessive consumption of ethanol [3]. The initial statements that 70–90% of cases of chronic pancreatitis are attributable to alcohol consumption has been recently challenged by studies from the United States, Italy and Denmark, and currently it appears that alcohol causes about 50% of chronic pancreatitis [4]; In Brazil, a study by Dani *et al*. reported that chronic alcoholism was responsible for 89.6% of cases [5]. Patterns of alcohol consumption in the Brazilian population were investigated in a survey conducted by Laranjeira *et al*. [6]; it was observed that 9% of the population drank ethanol abusively, defined as greater than 50 g of ethanol at least once a week. The risk for developing pancreatic disease occurs when there is a daily consumption of greater than 100 g of ethanol for men and 80 g for women for more than five years; therefore, less than 9% of the Brazilian population is at risk for developing this disease. There are few studies on the prevalence of chronic pancreatitis; they include those in Copenhagen in 1979, which reported a prevalence of 27.4 cases per 100,000 inhabitants [7] and a study in Japan, which found a prevalence of 28.9 cases per 100,000 inhabitants in 1994 [8]. It is difficult to transpose these results to other populations, since the risk factors vary in different countries and regions [9].

Despite that excessive consumption of ethanol is primarily responsible for most cases of chronic pancreatitis, alcohol alone is not sufficient to lead to the disease; in fact, only a small proportion of chronic alcoholics (5–10%) have the disease. The role of environmental cofactors, one’s genetic profile, or their interaction in determining the susceptibility to alcoholic chronic pancreatitis is still poorly understood [9]. Some environmental risk factors have been studied, and of these, smoking proven to be independently associated chronic pancreatitis [4]. After all pancreatic disease is a complex disorder resulting from multiple defects, which, when combined, lead to failure of control systems and metabolic homeostasis. The determination of the factors, genes, proteins, and cells involved; the pathways that govern its interactions; and where the defects are will provide a better understanding of the pathogenesis of the disease [10]. More than one theory attempts to explain the mechanisms responsible for the development of pancreatitis of alcoholic etiology [11]. The first, based on the study of pancreatic histology, shows the formation of ductal closures, secondary to increased protein content in pancreatic juice, is the change that leads to obstruction, fibrosis, and calcification. Most patients already have some degree of parenchymal injury when presenting with the first acute crisis [3]. The second theory holds that acinar cell injury is secondary to the toxic effect of ethanol, which leads to an inflammatory process that culminates in fibrosis. This hypothesis is based on metabolites of alcohol, such as ethyl esters of fatty acids, causing depletion of adenosine triphosphate (ATP) and loss of calcium regulation, leading to mitochondrial damage and increased susceptibility to activation of intra pancreatic trypsinogen [12]. Alcohol also leads to up regulation of systems that mediate the production of cytokines and other pro-inflammatory molecules (protein kinase C, nuclear factor κB, activator protein 1, etc.) and generate reactive oxygen species (ROS) [13]. Thus, a sentinel event would encourage infiltration of chronic inflammatory cells, activation of pancreatic stellate cells, and, therefore, fibrosis [12]. Accordingly, acute and chronic pancreatitis may not be independent entities, as characteristics of both often coincide; rather, both could be part of the spectrum of manifestations of the same disease [14].

## 2. The Role of Genetics

In this sense, molecular biology has contributed greatly to the understanding of the pathogenesis of pancreatitis. In 1996, Whitcomb *et al*. found the R122H mutation in the cationic trypsinogen gene (*PRSS1*) was associated with hereditary pancreatitis [15]. This study also showed inflammation was definitively linked to pancreatic enzyme activation and autodigestion of the parenchyma, as proposed by Chiari in 1896 [16]. Evidence suggests mutations in *PRSS1* cause the prematurely activated trypsin to be resistant to inactivation through autolysis, since arginine residues in the peptide chain are known sites of hydrolysis by trypsin itself. In the R122H mutation, arginine is substituted with histidine at codon 122, located on the external surface of the cationic trypsinogen molecule; this leads to inappropriate activity of trypsin, which damages the acinar cells and leads to ductal and interstitial injury through direct activation of other digestive zymogens and immunogenic stimuli [17].

Hereditary pancreatitis is characterized by an early onset in childhood, repeated bouts of acute pancreatitis, progression to fibrosis and pancreatic insufficiency, and high risk of adenocarcinoma of the pancreas, about 50 times higher than the general population [18,19]. It is a rare disease transmitted as an autosomal dominant trait, with a penetrance of about 80%. The diagnostic criteria includes a family history of the patient; for the diagnosis, there must be recurrent acute pancreatitis or chronic pancreatitis in at least two first degree relatives or three or more second degree relatives in two or more generations, for whom there were no identified precipitating factors. Since they are rigorous, these criteria have been questioned; some institutions define hereditary pancreatitis as any patient with no other detectable cause for the disease and a relative of first or second degree with chronic pancreatitis. The search for genetic mutations has also been adopted as a diagnostic tool [20,21]. In addition to R122H, which is the most frequent mutation in these patients, the N29I mutation was found to be the second most frequent; others were also identified, including A16V, D22G, K23R, N29T, E79K, and R122C [22–29].

Surveys were performed to attempt to correlate the development of alcoholic chronic pancreatitis with *PRSS1* gene mutations. Monaghan *et al*. in the USA, Perri *et al*. in Italy, and Chandak *et al*. in India studied these populations, but found no mutations [30–32]. In Brazil, Bernardino *et al*. [33] identified the E79K mutation in exon 3 of the *PRSS1* gene in only one out of 64 patients with alcoholic pancreatitis and there was no statistically significant difference when compared to the control group. Therefore, as expected, a significant association between alcoholic pancreatitis and *PRSS1* gene mutations was not demonstrated since the presence of these mutations in affected families causes early presenting pancreatic disease, where its manifestation depends little on the interference of other factors [1,34].

Moreover, there are protection mechanisms directed against pancreatic activation of trypsinogen and prevention of sustained activity of trypsin [10]. The *SPINK1* (serine protease inhibitor Kazal type 1) gene encodes a protein inhibitor of trypsin, which is synthesized in acinar cells and is able to inhibit 20% of intracellular activity of trypsin. It is considered the first line of defense against accidental premature activation of trypsinogen within acinar cells [35]. In 2000, the N34S mutation in exon 3 of *SPINK1* was identified in an analysis of patients with pancreatitis and no mutations in the *PRSS1* gene [36]. Further studies determined there is an association between this mutation and pancreatitis. A meta-analysis performed by Aoun *et al*. [37] included 24 case-control studies that investigated the frequency of this mutation in their populations; it was concluded that the N34S mutation is strongly associated with idiopathic, tropical, and alcoholic chronic pancreatitis, but with less impact on the latter. Other mutations, in addition to N34S, were found in the *SPINK1* gene, such as P55S, M1T, L14P, and others, but at much lower frequencies [38].

Chymotrypsin C (CTRC) is also responsible for the degradation of trypsin and trypsinogen isoforms with high specificity. Mutations in the *CTRC* encoding gene are potentially new candidates associated with pancreatic diseases [39]. In the study conducted by Rosendahl *et al*. [39] in 2008, the R254W mutation in exon 7 of the *CTRC* gene was found in 2.3% of 348 patients with alcoholic pancreatitis and in 0.5% of 432 patients with chronic liver disease caused by ethanol consumption, the difference being statistically significant (P = 0.03, OR = 5.1, 95% CI = 1.1–24.0). Chang *et al*. [40] identified other variants and haplotypes of the *CTRC* gene in a population of 126 patients with chronic pancreatitis of different etiologies, also occurring at a rate of 2.3%.

The protection of ductal cells depends on maintaining an alkaline pH and a rapid enzyme flow into the duodenum, which requires normal functioning of CFTR (cystic fibrosis transmembrane conductance regulator or transmembrane conductance regulator Cystic Fibrosis) [10]. This protein is present on the surface of the majority of human epithelial cells and functions as a chloride channel also responsible for the secretion of bicarbonate to pancreatic juices. In 1989, cystic fibrosis, an autosomal recessive disease characterized by severe lung injury, liver disease, atresia of the vas deferens, and pancreatic insufficiency manifested since childhood was attributed to mutations in this gene. Since then, more than 1000 mutations have been described in the *CFTR* gene and the disease was shown to have a much more varied phenotypic spectrum that can occur as monosymptomatic or atypical forms, such as the development of isolated pancreatic disease, secondary to mutations that reduce but do not completely eliminate the function of the protein [41]. It has also been suggested that mutations in the *CFTR* gene could predispose individuals to alcoholic chronic pancreatitis. It is difficult to test this hypothesis because it is a long gene comprising 24 exons that together encode a single protein of 1480 amino acids [42]. Table 1 lists some of the research completed so far [43–56].

Despite the high number of studies performed, it is difficult to reach a definitive conclusion about the real impact of carrying mutations in this gene on determining the susceptibility to alcoholic pancreatitis. This is due to several factors, including the variability in the methodologies, the number of mutations studied, and the number of patients included, which was most often inadequate and did not include a control population. In two studies conducted in Brazil, the methodology used was different. In the first study, Bernardino *et al*. [33] included patients with pancreatitis of other etiologies in addition to patients with pancreatic diseases due to alcohol and used a technique known as SSCP (Single Strand Conformational Polymorphism) to screen for mutations. Regions with found abnormalities were subsequently sequenced. This allowed the detection of mutations in the *CFTR* gene in five of 16 patients with idiopathic pancreatitis and in three of 64 patients with chronic pancreatitis caused by ethanol abuse. Yet, the presence of mutations in the *CFTR* gene was not investigated in the control population.

We performed the second study (da Costa *et al*. [56]). Intron 8 of the *CFTR* gene was studied by sequencing in chronic alcoholics diagnosed with pancreatic disease (group A), alcoholics without pancreatitis (group B), and blood donor volunteers who served as healthy controls (Group C). Different genetic profiles between the populations were found, with the 5T/7T genotype found more frequently (11.8% of cases, P < 0.05) in group A than in alcoholics without pancreatitis (group B, 2.9% of cases). The presence of a combination of haplotypes, (TG) 10-T7/(TG) 11-T7, occurred more frequently (P < 0.05) in controls (groups B and C; 23.5% and 20.2%, respectively) than in patients (group A; 7.3%).

In addition to the previously mentioned studies, other research was conducted on the occurrence of mutations in several other genes. In patients with pancreatitis of alcoholic etiology, the hypothesis remained that mutations would act as cofactors that influence an individual’s susceptibility, predisposing them to develop the disease in the presence of the aggressive environmental factor, alcohol [2]. Other genes that may be involved in the pathogenesis of alcoholic pancreatitis include those that code for enzymes that act in the metabolism of ethanol which, once absorbed, is catalyzed in the liver initially to acetaldehyde and then to acetate. The enzymes predominantly involved in this process include alcohol dehydrogenase and aldehyde dehydrogenase [13]. Kimura *et al*. [57]; Shimosegawa, Kume, and Masamune [58]; and Cichoz-Lach *et al*. [59] found different polymorphisms that varied between the studies. However, no definite conclusions were drawn about the participation of these genes in the development of alcoholic pancreatitis.

Another candidate gene is *CASR* (calcium sensing receptor). Hypercalcemia is associated with pancreatitis, possibly through the activation of trypsinogen and stabilization of trypsin [60]. CASR has a role in the homeostasis of calcium and was identified in both ductal and acinar cells [61]. In a study directed by Muddana *et al*. [62], an association was found (P = 0.018) between the R990G mutation and pancreatic diseases in patients who reported moderate or high ethanol consumption. Moreover, studies investigated the frequency of polymorphisms in genes encoding cytokines such as interleukins, TNFα (tumor necrosis factor–alpha), TGFβ1 (transforming growth factor–beta–1), INFδ (interferon–gamma), VEGF (vascular endothelial growth factor), and ICAM-1 (inter-cellular adhesion molecule 1). These cytokines are important in the development of inflammation and establishment of fibrosis; yet, results were variable and a no clear role was found for determining predisposition to the disease [63–65]. Other studies evaluated genes encoding detoxified enzymes such as glutathione S transferase [66–68], UDP glucuronosyltransferase [69], manganese - superoxide dismutase [66], and methylenetetrahydrofolate reductase [70]. These enzymes provide protection against products of oxidative stress, which apparently contribute to the development of pancreatitis [71]. While some researchers [66,67] found no association with chronic pancreatic disease, others [68–70] suggest the presence of low levels of detoxification may be a risk factor. The relevance of genetic variations leading to deficiencies of serum antiproteases, such as alpha 1 antitrypsin and alpha 2 macroglobulin, remains speculative [72,73].

## 3. Conclusions

In summary, we conclude that alcoholic pancreatitis is a complex disorder. The combination of, or interaction between, alcohol consumption and genetic factors results in the disease; yet, further studies are needed to improve the understanding of the mechanisms governing these overlapping causes. Furthermore, it is crucial to understand the profile of the population receiving health care. Brazil is characterized by multiple ethnic and racial mixes and is highly variable between regions. This may have implications in the approach for diagnosing patients, possibly leading to individualized follow-up and treatment.

## Figures and Tables

**Table 1:** Comparison of studies investigating mutations in the CFTR gene in patients with alcoholic pancreatitis (AP).

Authors	Year	Country	N		Number or type of mutations studied	Frequency (%) of *CFTR* mutations found	
			AP	Controls		AP	Controls
Sharer *et al*. [43]	1998	England	71	600	22	8.5%	* 5.3%
Arduino *et al*. [44]	1999	Italy	19	-	12	5.3%	-
Harber *et al*. [45]	1999	Australia	52	50	5T allele	3.9%	14%
Kimura *et al*. [46]	2000	Japan	31	47	ΔF508, R117H, 5T	9.7%	0%
Monaghan *et al*. [30]	2000	USA	46	-	40	4.4%	-
Malats *et al*. [47]	2001	Spain	76	-	ΔF508 e 5T	10.5%	-
Truninger *et al*. [48]	2001	Switzerland	49	-	31	10.2%	-
Gaia *et al*. [49]	2002	Italy	21	-	DGGE screening	0%	-
Bernardino *et al*. [33]	2003	Brazil	64	-	SSCP screening	4.7%	-
Perri *et al*. [31]	2003	Italy	45	34	31	8.9%	3.2%
Pezzilli *et al*. [50]	2003	Italy	34	-	29	23.5%	-
Casals *et al*. [51]	2004	Spain	37	-	DGGE/ SSCP screening	40.5%	-
Fujiki *et al*. [52]	2004	Japan	51	162	29	3.9%	1.2%
Naruse *et al*. [53]	2004	Japan	21	25	29	0%	0%
Lee *et al*. [54]	2005	Korea	43	35	22	2.4%	0%
Zoller *et al*. [55]	2007	Austria	24	-	29	8.3%	-
da Costa *et al*. [56]	2009	Brazil	68	68	5T/7T genotype	11.8%*	2.9%

* P < 0.05 = statistically significant; DGGE, denaturing gradient gel electrophoresis.

## References

[1] Behrman SW, Fowler ES (2007). Pathophysiology of chronic pancreatitis. Surg. Clin. N. Am.

[2] Apte MV, Wilson JS (2003). Alcohol-induced pancreatic injury. Best Pract. Res. Clin. Gastroenterol.

[3] Lucrezio L, Bassi M, Migliori M, Bastagli L, Gullo L (2008). Alcoholic pancreatitis. New pathogenetic insights. Minerva Med.

[4] Yadav D, Whitcomb DC (2010). The role of alcohol and smoking in pancreatitis. Nat. Rev. Gastroenterol. Hepatol.

[5] Dani R, Mott CB, Guarita DR, Nogueira CE (1990). Epidemiology and etiology of chronic pancreatitis in Brazil: a tale of two cities. Pancreas.

[6] Laranjeira R, Pinsky I, Zaleski M, Caetano R (2007). 1º Levantamento Nacional sobre os padrões de consumo de álcool na população brasileira.

[7] Copenhagen Pancreatitis Study (1981). An interim report from a prospective epidemiological multicentric study. Scand J. Gastroenterol.

[8] Lin Y, Tamakoshi A, Matsuno S, Takeda K, Hayakawa T, Kitagawa M, Naruse S, Kawamura T, Wakai K, Aoki R (2000). Nationwide epidemiological survey of chronic pancreatitis in Japan. J. Gastroenterol.

[9] Yadav D, Papachristou GI, Whitcomb DC (2007). Alcohol-associated pancreatitis. Gastroenterol. Clin. N. Am.

[10] Whitcomb DC, Barmada MM (2007). A systems biology approach to genetic studies of pancreatitis and other complex diseases. Cell. Mol. Life Sci.

[11] Hayakawa T, Naruse S, Kitagawa M, Ishiguro H, Jin CX, Kondo T (2002). Clinical evidence of pathogenesis in chronic pancreatitis. J. Hepatobiliary Pancreat. Surg.

[12] Whitcomb DC (2006). Gene–environment factors that contribute to alcoholic pancreatitis in humans. J. Gastroenterol. Hepatol.

[13] Pandol SJ, Raraty M (2007). Pathobiology of alcoholic pancreatitis. Pancreatology.

[14] Mariani A, Testoni PA (2008). Is acute recurrent pancreatitis a chronic disease?. World J. Gastroenterol.

[15] Whitcomb DC, Gorry MC, Preston RA, Furey W, Sossenheimer MJ, Ulrich CD, Martin SP, Gates LK, Amann ST, Toskes PP (1996). Hereditary pancreatitis is caused by a mutation in the cationic trypsinogen gene. Nat. Genet..

[16] Chiari H (1896). Ueber Selbstverdauung des menschlichen Pankreas. Zeitschrift für Heilkunde.

[17] Whitcomb DC (2000). Genetic predispositions to acute and chronic pancreatitis. Med. Clin. N. Am.

[18] Rebours V, Boutron-Ruault MC, Schnee M, Férec C, Le Maréchal C, Hentic O, Maire F, Hammel P, Ruszniewski P, Lévy P (2009). The natural history of hereditary pancreatitis: A national series. Gut.

[19] Rosendahl J, Bödeker H, Mössner J, Teich N (2007). Hereditary chronic pancreatitis. Orphanet J. Rare Dis.

[20] Rebours V, Boutron-Ruault MC, Schnee M, Férec C, Maire F, Hammel P, Ruszniewski P, Lévy P (2008). Risk of pancreatic adenocarcinoma in patients with hereditary pancreatitis: A national exhaustive series. Am. J. Gastroenterol.

[21] Teich N, Mössner J (2008). Hereditary chronic pancreatitis. Best Pract. Res. Clin. Gastroenterol.

[22] Gorry MC, Gabbaizedeh D, Furey W, Gates LK, Preston RA, Aston CE, Zhang Y, Ulrich C, Ehrlich GD, Whitcomb DC (1997). Mutations in the cationic trypsinogen gene are associated with recurrent acute and chronic pancreatitis. Gastroenterology.

[23] Teich N, Mössner J, Keim V (1998). Mutations of the cationic trypsinogen in hereditary pancreatitis. Hum. Mutat.

[24] Ferec C, Raguénès O, Salomon R, Roche C, Bernard JP, Guillot M, Quere I, Faure C, Mercier C, Audrezet M (1999). Mutations in the cationic trypsinogen gene and evidence for genetic heterogeneity in hereditary pancreatitis. J. Med. Genet.

[25] Witt H, Luck W, Becker M (1999). A signal peptide cleavage site mutation in the cationic trypsinogen gene is strongly associated with chronic pancreatitis. Gastroenterology.

[26] Le Maréchal C, Chen JM, Quéré I, Raguénès O, Férec C, Auroux J (2001). Discrimination of three mutational events that result in a disruption of the R122 primary autolysis site of the human cationic trypsinogen (*PRSS1*) by denaturing high performance liquid chromatography. BMC Genet.

[27] Pfützer R, Myers E, Applebaum-Shapiro S, Finch R, Ellis I, Neoptolemos J, Kant JA, Whitcomb DC (2002). Novel cationic trypsinogen (*PRSS1*) N29T and R122C mutations cause autosomal dominant hereditary pancreatitis. Gut.

[28] Simon P, Weiss FU, Sahin-Toth M, Parry M, Nayler O, Lenfers B, Schnekenburger J, Mayerle J, Domschke W, Lerch MM (2002). Hereditary pancreatitis caused by a novel *PRSS1* mutation (Arg-122 --> Cys) that alters autoactivation and autodegradation of cationic trypsinogen. J. Biol. Chem.

[29] Teich N, Le Maréchal C, Kukor Z, Caca K, Witzigmann H, Chen JM, Tóth M, Mössner J, Keim V, Férec C (2004). Interaction between trypsinogen isoforms in genetically determined pancreatitis: mutation E79K in cationic trypsin (*PRSS1*) causes increased transactivation of anionic trypsinogen (*PRSS2*). Hum. Mutat.

[30] Monaghan KG, Jackson CE, KuKuruga DL, Feldman GL (2000). Mutation analysis of the cystic fibrosis and cationic trypsinogen genes in patients with alcohol-related pancreatitis. Am. J. Med. Genet.

[31] Perri F, Piepoli A, Stanziale P, Merla A, Zelante L, Andriulli A (2003). Mutation analysis of the cystic fibrosis transmembrane conductance regulator (*CFTR*) gene, the cationic trypsinogen (*PRSS1*) gene, and the serine protease inhibitor, Kazal type 1 (*SPINK1*) gene in patients with alcoholic chronic pancreatitis. Eur. J. Hum. Genet.

[32] Chandak GR, Idris MM, Reddy DN, Mani KR, Bhaskar S, Rao GV, Singh L (2004). Absence of *PRSS1* mutations and association of *SPINK1* trypsin inhibitor mutations in hereditary and non-hereditary chronic pancreatitis. Gut.

[33] Bernardino AL, Guarita DR, Mott CB, Pedroso MR, Machado MC, Laudanna AA, Tani CM, Almeida FL, Zatz M (2003). *CFTR, PRSS1* e *SPINK1* mutations in the development of pancreatitis in Brazilian patients. JOP. J. Pancreas (Online).

[34] Whitcomb DC (2003). Genetic predisposition to alcoholic chronic pancreatitis. Pancreas.

[35] Weiss FU, Simon P, Mayerle J, Kraft M, Lerch MM (2006). Germline mutations and gene polymorphism associated with human pancreatitis. Endocrinol. Metab. Clin. N. Am.

[36] Chen JM, Mercier B, Audrezet MP, Ferec C (2000). Mutational analysis of the human pancreatic secretory trypsin inhibitor (*PSTI*) gene in hereditary and sporadic chronic pancreatitis. J. Med. Genet.

[37] Aoun E, Chang CC, Greer JB, Papachristou GI, Barmada MM, Whitcomb DC (2008). Pathways to injury in chronic pancreatitis: decoding the role of the high-risk *SPINK1* N34S haplotype using meta-analysis. PLoS ONE.

[38] Drenth JPH, Morsche R, Jansen JBMJ (2002). Mutations in serine protease inhibitor Kazal type 1 are strongly associated with chronic pancreatitis. Gut.

[39] Rosendahl J, Witt H, Szmola R, Bhatia E, Ozsvári B, Landt O, Schulz HU, Gress TM, Pfützer R, Löhr M (2008). Chymotrypsin C (*CTRC*) alterations that diminish activity or secretion are associated with chronic pancreatitis. Nat. Genet.

[40] Chang MC, Chang YT, Wei SC, Liang PC, Jan IS, Su YN, Kuo CH, Wong JM (2009). Association of novel chymotrypsin C gene variations and haplotypes in patients with chronic pancreatitis in Chinese in Taiwan. Pancreatology.

[41] Witt H, Becker M (2002). Genetics of chronic pancreatitis. J. Pediatr. Gastroenterol. Nutr.

[42] Hank C, Schneider A, Whitcomb DC (2003). Genetic polymorphisms in alcoholic pancreatitis. Best Pract. Res. Clin. Gastroenterol.

[43] Sharer N, Schwarz M, Malone G, Howarth A, Painter J, Super M, Braganza J (1998). Mutations of the cystic fibrosis gene in patients with chronic pancreatitis. N. Engl. J. Med.

[44] Arduino C, Gallo M, Brusco A, Garnerone S, Piana MR, Di Maggio S, Gerbino Promis G, Ferrone M, Angeli A (1999). Polyvariant mutant CFTR genes in patients with chronic pancreatitis. Clin. Genet.

[45] Harber PS, Norris MD, Apte MV, Rodgers SC, Norton ID, Pirola RC, Roberts-Thomson IC, Wilson JS (1999). Alcoholic pancreatitis and polymorphisms of the variable length polythymidine tract in the cystic fibrosis gene. Alcohol. Clin. Exp. Res.

[46] Kimura S, Okabayashi Y, Inushima K, Yutsudo Y, Kasuga M (2000). Polymorphism of cystic fibrosis gene in Japanese patients with chronic pancreatitis. Dig. Dis. Sci.

[47] Malats N, Casals T, Porta M, Guarner L, Estivill X, Real FX (2001). Cystic fibrosis transmenbrane regulator (*CFTR*) ΔF508 mutation and 5T allele in patients with chronic pancreatitis and exocrine pancreatic cancer. Gut.

[48] Truninger K, Malik N, Ammann RW, Muellhaupt B, Seifert B, Müller HJ, Blum HE (2001). Mutation of the cystic fibrosis gene in patients with chronic pancreatitis. Am. J. Gastroenterol.

[49] Gaia E, Salacone P, Gallo M, Promis GG, Brusco A, Bancone C, Carlo A (2002). Germline mutations in *CFTR* and *PSTI* genes in chronic pancreatitis patients. Dig. Dis. Sci.

[50] Pezzilli R, Morselli-Labate AM, Mantovani V, Romboli E, Selva P, Migliori M, Corinaldesi R, Gullo L (2003). Mutations of the *CFTR* gene in pancreatic disease. Pancreas.

[51] Casals T, Aparisi L, Martínez-Costa C, Giménez J, Ramos MD, Mora J, Diaz J, Boadas J, Estivill X, Farré A (2004). Different *CFTR* mutation spectrum in alcoholic and idiopathic chronic pancreatitis?. Pancreas.

[52] Fujiki K, Ishiguro H, Ko SB, Mizuno N, Suzuki Y, Takemura T, Yamamoto A, Yoshikawa T, Kitagawa M, Hayakawa T (2004). Genetic evidence for *CFTR* dysfunction in Japanese: background for chronic pancreatitis. J. Med. Genet..

[53] Naruse S, Ishiguro H, Suzuki Y, Fujiki K, Ko SB, Mizuno N, Takemura T, Yamamoto A, Yoshikawa T, Jin C (2004). A finger sweat chloride test for the detection of a high-risk group of chronic pancreatitis. Pancreas.

[54] Lee KH, Ryu JK, Yoon WJ, Lee JK, Kim YT, Yoon YB (2005). Mutation analysis of *SPINK1* and *CFTR* gene in Korean patients with alcoholic chronic pancreatitis. Dig. Dis. Sci.

[55] Zoller H, Egg M, Graziadei I, Creus M, Janecke AR, Löffler-Ragg J, Vogel W (2007). *CFTR* gene mutations in pancreatitis: Frequency and clinical manifestations in an Austrian patient cohort. Wien Klin. Wochenschr.

[56] da Costa MZ, Guarita DR, Ono-Nita SK, Nogueira JdeA, Nita ME, Paranaguá-Vezozzo DC, de Souza MT, do Carmo EP, Teixeira AC, Carrilho FJ (2009). *CFTR* polymorphisms in patients with alcoholic chronic pancreatitis. Pancreatology.

[57] Kimura S, Okabayashi Y, Inushima K, Kochi T, Yutsudo Y, Kasuga M (2000). Alcohol and aldehyde dehydrogenase polymorphisms in Japanese patients with alcohol-induced chronic pancreatitis. Dig. Dis. Sci.

[58] Shimosegawa T, Kume K, Masamune A (2008). *SPINK1*, *ADH2*, and *ALDH2* gene variants and alcoholic chronic pancreatitis in Japan. J. Gastroenterol. Hepatol.

[59] Cichoz-Lach H, Partycka J, Nesina I, Celinski K, Słomka M, Wojcierowski J (2006). Genetic polymorphism of alcohol dehydrogenase 3 in alcohol liver cirrhosis and in alcohol chronic pancreatitis. Alcohol. Alcohol.

[60] Etemad B, Whitcomb DC (2001). Chronic pancreatitis: Diagnosis, classification, and new genetic developments. Gastroenterology.

[61] Bruce JI, Yang X, Ferguson CJ, Elliott AC, Steward MC, Case RM, Riccardi D (1999). Molecular and functional identification of a Ca2+ (polyvalent cation)-sensing receptor in rat pancreas. J. Biol. Chem.

[62] Muddana V, Lamb J, Greer JB, Elinoff B, Hawes RH, Cotton PB, Anderson MA, Brand RE, Slivka A, Whitcomb DC (2008). Association between calcium sensing receptor gene polymorphisms and chronic pancreatitis in a US population: Role of serine protease inhibitor Kazal 1type and alcohol. World J. Gastroenterol.

[63] Howell WM, Pead PJ, Shek FW, Rose-Zerilli MJ, Armstrong T, Johnson CD, Fine DR, Iredale JP, Bateman AC (2005). Influence of cytokine and ICAM1 gene polymorphisms on susceptibility to chronic pancreatitis. J. Clin. Pathol.

[64] Talar-Wojnarowska R, Gasiorowska A, Smolarz B, Romanowicz-Makowska H, Kulig A, Malecka-Panas E (2009). Clinical significance of interleukin-6 (Il-6) gene polymorphism and Il-6 serum level in pancreatic adenocarcinoma and chronic pancreatitis. Dig. Dis. Sci.

[65] Bendicho MT, Guedes JC, Silva NN, Santana GO, dos Santos RR, Lyra AC, Lyra LG, Meyer R, Lemaire DC (2005). Polymorphism of cytokine genes (TGF – [beta] 1, IFN – [gamma], IL-6, IL-10, and TNF – [alfa]) in patients with chronic pancreatitis. Pancreas.

[66] Österreicher CH, Schultheiss J, Wehler M, Homann N, Hellerbrand C, Künzli B, Friess H, Seitz HK, Stickel F (2007). Genetic polymorphisms of manganese-superoxide dismutase and glutathione-S-transferase in chronic alcoholic pancreatitis. Mutagenesis.

[67] Schneider A, Tögel S, Barmada MM, Whitcomb DC (2004). Genetic analysis of the glutathione s-transferase genes *MGST1*, *GSTM3*, *GSTT1*, and *GSTM1* in patients with hereditary pancreatitis. J. Gastroenterol.

[68] Verlaan M, te Morsche RH, Roelofs HM, Laheij RJ, Jansen JB, Peters WH, Drenth JP (2003). Glutathione S-transferase Mu null genotype affords protection against alcohol induced chronic pancreatitis. Am. J. Med. Genet.

[69] Ockenga J, Vogel A, Teich N, Keim V, Manns MP, Strassburg CP (2003). UDP Glucuronosyltransferase (UGT1A7) gene polymorphisms increase the risk of chronic pancreatitis and pancreatic cancer. Gastroenterology.

[70] Nisevic I, Dinic J, Nikolic A, Djordjevic V, Lukic S, Ugljesic M, Andjelic-Jelic M, Petrovic-Stanojevic N, Radojkovic D (2008). *MTHFR C677T* polymorphism in chronic pancreatitis and pancreatic adenocarcinoma. Cell Biochem. Funct.

[71] Braganza JM (1998). A framework for the aetiogenesis of chronic pancreatitis. Digestion.

[72] Sobczynska-Tomaszewska A, Bak D, Oralewska B, Oracz G, Norek A, Czerska K, Mazurczak T, Teisseyre M, Socha J, Zagulski M (2006). Analysis of *CFTR, SPINK1, PRSS1* and *AAT* mutations in children with acute or chronic pancreatitis. J. Pediatr. Gastroenterol. Nutr.

[73] Teich N, Walther K, Bödeker H, Mössner J, Keim V (2002). Relevance of variants in serum antiproteinases for the course of chronic pancreatitis. Scand. J. Gastroenterol.

